# Effects of Alcoholic Liquors in Phthisis

**Published:** 1857-01

**Authors:** 


*Effects of Alcoholic Liquors in Phthisis.*

A writer (Prof. Lawson, we presume from the initials,) in the December number of the *Western Lancet*, pays us the compliment of devoting eight pages to a review of the Address on the above subject, which we had the pleasure of reading to the Members of the Illinois State Medical Society at their last annual meeting.

The reviewer thinks, that, in drawing inferences from our experiments, we have committed the grave error of forming 'conclusions from the *Pathological* instead of the *Therapeutical* effects of alcohol.' Will he be kind enough to give us the symptoms or landmarks by which we may know where the *Therapeutical* effects end and the *Pathological* begin?

Dr. Bocker, in his experiments, used only one *tea-spoonful of spirits of wine* at a time, and yet the results he obtained correspond in all important particulars with those obtained by me. Will the reviewer tell us what constitutes the maximum quantity for a *Therapeutic* effect on a man of ordinary size and health? In reference to our position, that alcohol is not a *tonic* or invigorating agent, the reviewer says:—'If the author of the Address will look at the *rubicund* countenance, *red nose*, *florid complexion*, and active circulation of the *dram*-drinker (before disease supervenes), he will hardly deny that alcoholic preparations possess very decided and potent *stimulating* and *sustaining* properties.' Let us reflect a little on this paragraph, and see if the reviewer has not confounded several things essentially distinct:—

*First*—What constitutes a test of the *tonic* or *invigorating* qualities possessed by any individual? Is it merely a *rubicund countenance, a red nose, and a florid complexion?* If so, then the red-faced, jolly, and aldermanic beer-drinker should present us the maximum of strength and vigor. But who does not know that the physical activity and power of endurance, possessed by such individuals, *diminish* precisely in the same ratio as they become more rubicund and plethoric? Can our friend of the *Lancet* find a single specimen of this kind, who, if put to the test of active physical exertion, will not soon demonstrate the fact that there is no direct ratio between the 'florid complexion' of the 'dram-drinker' and his actual strength and physical energy?

*Second*—What constitutes the dividing line between the 'rubicund countenance, red nose, and florid complexion of the dram-drinker' *before disease supervenes*, and the same *after* the commencement of the latter? May we safely presume disease to be absent so long as the nose is *red only*, and the reverse when the acnæ rosacæ begin to be added to the redness?

*Third*—Are the words, 'stimulating' and 'sustaining'—*stimulating* and invigorating—stimulant and tonic, merely synonymous with each other? In other words, is there no essential difference between a stimulant or excitant, and a tonic? If there is, under which head does alcohol belong?

Again, our reviewer says:—'Whatever difference of opinion may exist in regard to the effects of retained carbon, no *candid* and accurate observer can doubt the beneficial effects of alcoholic stimulants, when given in suitable cases and proper forms and doses, in *promoting primary* assimilation.'

We may not claim any special *accuracy* as an observer, but certain it is, that, after nearly twenty years of most careful observation, experiment, and reflection concerning this very subject, we *do doubt* the power of alcohol to promote 'primary assimilation.' Is Dr. L. M. L. quite certain that he has not mistaken the effects of simple *retarded* disintegration or transformation of tissues, for a positive *increase* of assimilation? He certainly will not deny that the alcohol, by its immediate and primary action, diminishes the exalation of carbon from the lungs and nearly all the other eliminations derived from the transformation of tissues, as proved by the experiments of Drs. Prout, Sandras, Bouchardet, Magnus, and Bocker, (without including our own.) Indeed he admits, in so many words, 'that it checks excessive transformation of the tissues.' Now, will Dr. L. be kind enough to explain by what physiological or therapeutic process any agent can positively *increase* primary assimilation, and at the same time *diminish* or hold in check the transformation of the tissues with the resulting eliminations?

Finally, our reviewer disposes of the thirty-seven cases, reported in the Address, in the following easy and summary manner:—'We are not able to perceive that these cases prove anything in the premises. It will be remarked that a majority of the patients were Irish; and with the well-known habits of a majority of such persons, who are usually ill-fed and much exposed, it is not surprising that the alcoholic liquors should prove ineffectual. Nay, more, it is quite probable, if not positively certain, that the want of substantial food will, under the effects of the liquor, be positively pernicious. Most of these persons drink liquors of the worst possible quality, and in an irregular manner; often excessively, so that great evils will usually follow.'

Does this paragraph exhibit a philosophical or even a legitimate mode of reasoning? Have these general intimations about the habits of the *Irish* people as a class any necessary application to the individual cases reported by me? Have they any application to the well-fed, well-clothed, well-protected girl who was supplied a cup of beer regularly with her meals for three years? or was the *cup of beer* an over-dose, producing 'pathological' instead of therapeutic effects? Have they any better application to the country hotel-keeper, whose case is detailed, and who, with plenty of good food, good clothes, a competence pecuniarily, made a *regular* habit of taking, daily, *moderate* quantities (but not to intoxication) of alcoholic liquors for ten or twelve years? Indeed, if any such general statements as those contained in the above paragraph were allowed to have influence in determining questions of a scientific nature, we might with much propriety ask those who claim alcohol as a prophylactic in phthisis, how it happens that the Irish people, who more universally than any other people in Christendom drink alcoholic beverages, at the same time furnish the highest numerical ratio of both typhus, and tuberculosis or consumption? But our queries are extending much beyond the space we designed, and we will only add, that the writer in the *Lancet* is entirely in error when he supposes that our positions in relation to the action of alcohol on the human system, are 'the result of ultra-temperance notions;' or that we seek to exclude it from the list of important remedial agents. On the contrary, our positions are the result of years of study, observation, and experimental research. So far from excluding alcohol from the list of remedies, our positions simply exclude it from the Materia *Alimentaria*, or list of wholesome beverages, and confine it rigidly to the Materia *Medica*, in precisely the same sense as we confine opium, castor oil, or ipecac. Nay, we go further, and by pointing out definitely the two-fold power of alcohol to *excite* or *exhilarate* the nervous system, and at the same time diminish organic changes in the tissues, we suggest clearly the indications it is calculated to fulfil in the treatment of disease. That these indications are much more limited than those generally assigned by the profession, we admit. But the question of their correctness we cheerfully submit to the test of time and criticism; only hoping that those who dispute them, will give the results numerically of actual cases, observations, and experiments, instead of mere general statements and opinions.

